# Soluble suppression of tumorigenicity 2 associated with major adverse cardiac events in children with myocarditis

**DOI:** 10.3389/fcvm.2024.1404432

**Published:** 2024-05-13

**Authors:** Tongtong Shi, Jing Ge, Shan Li, Yali Zhang

**Affiliations:** ^1^ Department of Cardiology, The Affiliated Xuzhou Children's Hospital of Xuzhou Medical University, Xuzhou, Jiangsu, China; ^2^ Department of Clinical Nutrition, The Affiliated Huai'an Hospital of Xuzhou Medical University and Huai'an Second People’s Hospital, Huai'an, Jiangsu, China; ^3^ Department of Oncology, The Affiliated Huai'an Hospital of Xuzhou Medical University and Huai'an Second People’s Hospital, Huai'an, Jiangsu, China

**Keywords:** myocarditis, soluble suppression of tumorigenicity 2, children, MACEs, biomarker

## Abstract

**Objective:**

Soluble suppression of tumorigenicity 2 (sST2) is associated with the prognosis of some cardiac diseases, but studies on sST2 and the prognosis of patients with myocarditis are rare. This study investigated the relationship between major adverse cardiovascular events (MACEs) and sST2 during hospitalization in pediatric patients with myocarditis.

**Methods:**

This was a single-center retrospective cohort study. A total of 252 patients aged ≤14 years diagnosed with myocarditis were enrolled. Events during the hospitalization were defined as MACEs (all-cause death > new heart failure > ventricular arrhythmia).

**Results:**

A total of 25 people had MACEs during their hospital stay. The mortality during hospitalization was 6/23 (26%) in patients with heart failure and 3/10 (30%) in patients with ventricular arrhythmias. After including these risk factors in a multivariate logistic regression analysis, NT-proBNP (OR 4.323; 95% CI, 2.433–7.679; *p* < 0.001) and sST2 (OR 1.020; 95% CI, 1.003–1.037; *p* = 0.022) remained statistically significant and were independent risk factors for MACEs during hospitalization in pediatric myocarditis patients.

**Conclusions:**

Elevated levels of NT-proBNP and sST2 were independently associated with major adverse cardiovascular events during hospitalization in children with myocarditis, and both showed good predictive efficacy.

## Introduction

Myocarditis is an inflammatory disease that causes necrosis or degeneration of cardiomyocytes (1), and it is a common cause of morbidity and mortality in pediatric patients (2). A national registry study from Europe showed that the incidence of acute myocarditis in children is as high as 1.95 per 100,000 per year and is still rising (3, 4). Previous studies have shown that the mortality rate in children with myocarditis is between 7% and 17%, and the rate of need for mechanical circulatory support or heart transplantation can reach 30% (2). In children hospitalized for myocarditis, congestive heart failure and ventricular arrhythmias are common complications of myocarditis and are associated with increased mortality (5, 6). Therefore, exploring more predictors for major adverse cardiovascular events (MACEs) in children with myocarditis would be valuable.

It is well known that the development of myocarditis is closely related to inflammation (7, 8). Soluble suppression of tumorigenicity 2 (sST2) acts as a decoy receptor that promotes the inflammatory response by binding to interleukin (IL)-33 and inhibits the cardioprotective effects of IL-33 (9–12). sST2 is now considered a valuable prognostic and monitoring tool, and it was included in the American Heart Association's updated heart failure guidelines (13). Indeed, sST2 is prognostically relevant in a variety of diseases. Van Vark et al. showed an association between sST2 and all-cause mortality in a 1-year follow-up of 496 patients with acute heart failure (14). In patients with acute myocardial infarction, sST2 levels within 24 h were considered an early marker of prognosis (15). In patients with acute stroke, sST2 was an independent predictor of poor prognosis and all-cause mortality within the first 12 months (16). In a recent study, Coronado MJ et al. found higher sST2 concentrations in patients with myocarditis and that sST2 levels were associated with exacerbating heart failure symptoms (17). However, no studies on sST2 and prognosis in children with myocarditis have been reported. This study investigated the relationship between MACEs and sST2 during hospitalization in pediatric patients with myocarditis.

## Methods

### Study population

This was a single-center retrospective cohort study. We consecutively enrolled pediatric patients (aged ≤14 years) diagnosed with myocarditis (18) at the Xuzhou Children's Hospital from December 2020 to December 2023, and all patients were perfected sST2 detection. The study protocol was verified by the Xuzhou Children's Hospital Ethics Committee, and the written informed consent was exempted due to low risk to patients according to the relevant IRB regulatory guidelines. All patient details have been de-identified. The reporting of this study conforms to STROBE guidelines (19). Patients with known congenital or acquired heart disease before admission were excluded.

### Clinical and laboratory data

All patients were collected clinical baseline data, including gender, age, and weight. Venous blood samples were collected on admission for laboratory analysis. High-sensitivity C-reactive protein (hs-CRP), N-terminal B-type natriuretic peptide (NT-proBNP), creatine kinase isoenzyme MB (CKMB), neutrophil/lymphocyte ratio (NLR), creatinine, urea, uric acid, alanine transaminase (ALT) and aspartate transaminase (AST) were detected. Left ventricular ejection fraction (LVEF) and left ventricular end-diastolic diameter (LVED) were obtained by cardiac ultrasound. The concentration of sST2 in blood samples was determined using an enzyme-linked immunosorbent assay kit (ELISA) (ElabScience Biotechnology, China).

### Major adverse cardiovascular events during hospitalization

Events during the hospitalization were major adverse cardiovascular events (MACEs, all-cause death > new heart failure > ventricular arrhythmia). New congestive heart failure was identified as the first episode of cardiac decompensation requiring intravenous diuretic therapy (20). Ventricular arrhythmias include ventricular tachycardia and ventricular fibrillation. Ventricular tachycardia was defined as a ventricular tachycardia lasting ≥30 s, or, although less than 30 s, the patient is hemodynamically unstable, requiring immediate termination of the tachycardia (21).

### Statistical analysis

SPSS 24.0 software was used for statistical analysis. Data conforming to a normal distribution were expressed as mean ± standard deviation (SD) and analyzed by an unpaired *t*-test. Not normally distributed data were expressed as median (interquartile range, IQR) and analyzed by a non-parametric test (Mann-Whiney *U*-test). Categorical variables were analyzed by the chi-square test. Univariate and multivariate regression analyses were used to identify risk factors for MACEs. Receiver operating characteristic (ROC) was used to assess the sensitivity and specificity of risk factors for MACEs. *P* < 0.05 was considered to be statistically significant.

## Results

### Characteristics of the patients with myocarditis

**Figure 1:**
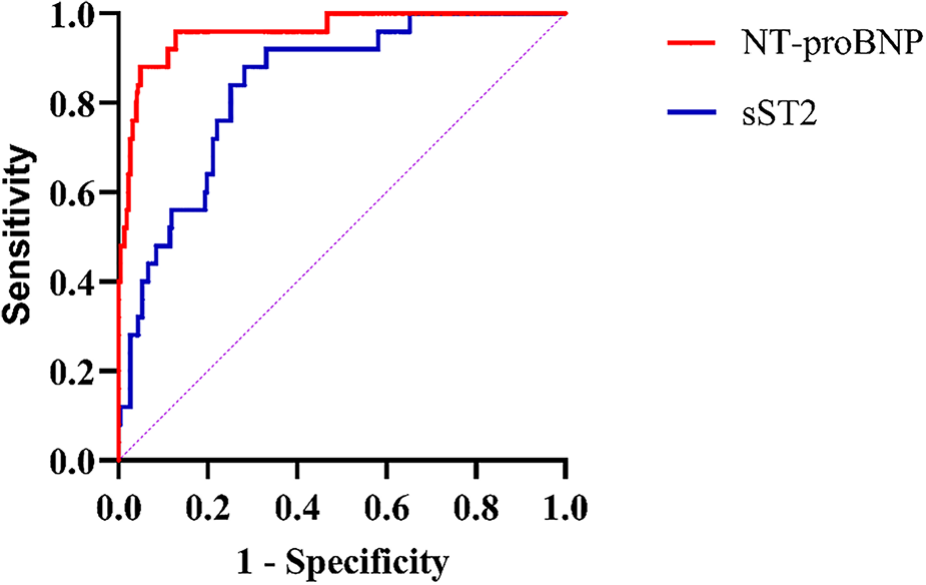
Mortality during hospitalization in patients combined with heart failure or ventricular arrhythmias.

A total of 252 patients were included in this study, with a male-to-female ratio of 1.2:1. A total of 25 people had MACEs during their hospital stay. Of these, 7 were all-cause deaths, 17 were heart failure, and 1 was ventricular arrhythmia. The mortality rate during hospitalization was 6/23 (26%) in patients with heart failure and 3/10 (30%) in patients with ventricular arrhythmias (Figure 1).

### Comparison of MACEs and no MACEs

Compared to the No MACEs group, patients in the MACEs group had significantly higher creatinine [32 (26, 39) vs. 42 (34, 50.5) umol/L; *p* < 0.001], urea [4.19 (3.37, 5.22) vs. 5.85 (4.91, 7.95) mmol/L; *p* < 0.001], AST [38 (27, 56) vs. 90 (62, 394) U/L; *p* < 0.001], ALT [18 (12, 28) vs. 38 (18, 133) U/L; *p* < 0.001], hs-CRP [1 (0.44, 3.86) vs. 5.67 (2.90, 17.24) mg/dl; *p* < 0.001], NLR [1.38 (0.82, 2.6) vs. 4.22 (2.09, 5.77); *p* < 0.001], NT-proBNP [141 (80, 460) vs. 4,710 (1,820, 12,200) pg/ml; *p* < 0.001], CKMB [45 (25, 89) vs. 61 (38.5, 131) ng/ml; *p* = 0.018], length of stay [6 (5, 7) vs. 10 (3, 14.5) days; *p* = 0.017], LVED [59 (56, 62) vs. 48 (35, 59.5) %; *p* < 0.001] and sST2 [19.69 (14.22, 27.22) vs. 47.18 (37, 82.13) ng/ml; *p* < 0.001], while LVEF [3.8 (3.4, 4.1) vs. 4.1 (3.6, 4.45) cm; *p* = 0.019] was significantly lower (*p* < 0.05) (Table 1).

**Table 1:** Patient characteristics.

	No MACEs (n=227)	MACEs (n=25)	*P*
Age, months	92 (48, 150)	108 (69.5, 142.5)	0.591
Male, *n* (%)	14 (56%)	121 (53.3%)	0.798
Weight, kg	21 (14, 34)	26.5 (8, 43.75)	0.068
Creatinine, umol/L	32 (26, 39)	42 (34, 50.5)	**<0** **.** **001**
Urea, mmol/L	4.19 (3.37, 5.22)	5.85 (4.91, 7.95)	**<0** **.** **001**
Uric acid, umol/L	282 (216, 368)	340 (235, 410.5)	0.099
AST, U/L	38 (27, 56)	90 (62, 394)	**<0** **.** **001**
ALT, U/L	18 (12, 28)	38 (18, 133)	**<0** **.** **001**
Hs-CRP, mg/dl	1 (0.44, 3.86)	5.67 (2.90, 17.24)	**<0** **.** **001**
NLR	1.38 (0.82, 2.6)	4.22 (2.09, 5.77)	**<0** **.** **001**
NT-proBNP, pg/ml	141 (80, 460)	4,710 (1,820, 12,200)	**<0** **.** **001**
CKMB, ng/ml	45 (25, 89)	61 (38.5, 131)	**0** **.** **018**
Length of stay, days	6 (5, 7)	10 (3, 14.5)	**0** **.** **017**
LVEF, %	59 (56, 62)	48 (35, 59.5)	**<0** **.** **001**
LVED, cm	3.8 (3.4, 4.1)	4.1 (3.6, 4.45)	**0** **.** **019**
sST2, ng/ml	19.69 (14.22, 27.22)	47.18 (37, 82.13)	**<0** **.** **001**

Hs-CRP, high-sensitivity C-reactive protein; NLR, neutrophil/lymphocyte ratio; AST, aspartate transaminase; ALT, alanine transaminase; NT-proBNP, N terminal pro-B-type natriuretic peptide; CKMB, creatine kinase isoenzyme MB; LVEF, left ventricular ejection fraction; LVED, left ventricular end diastolic; sST2, soluble suppression of tumorigenicity 2.

Bold values indicate significant *p* < 0.05.

### Univariate and multivariate logistic regression analysis

Univariate analysis found that creatinine, AST, ALT, hs-CRP, NT-proBNP, length of stay, LVED, sST2, and LVEF were associated with MACEs in pediatric myocarditis patients. After including these risk factors in a multivariate logistic regression analysis using a stepwise forward method, NT-proBNP (OR 4.323; 95% CI, 2.433–7.679; *p* < 0.001) and sST2 (OR 1.020; 95% CI, 1.003–1.037; *p* = 0.022) remained statistically significant and were independent risk factors for MACEs during hospitalization in pediatric myocarditis patients (Table 2).

**Table 2:** Univariate and multivariate logistic regression analysis.

	Univariate analysis	*P*	Multivariate analysis	*P*
	OR 95% CI		OR 95% CI	
Age, months	1.001 (0.993–1.010)	0.741		
Male, *n* (%)	0.897 (0.390–2.061)	0.798		
Weight, kg	1.015 (0.993–1.038)	0.177		
Creatinine, umol/L	1.048 (1.020–1.077)	**0** **.** **001**		
Urea, mmol/L	1.036 (0.986–1.089)	0.163		
Uric acid, umol/L	1.002 (1.000–1.005)	0.098		
AST, U/L	1.010 (1.006–1.015)	**<0** **.** **001**		
ALT, U/L	1.015 (1.005–1.026)	**0** **.** **003**		
Hs-CRP, mg/dl	1.030 (1.007–1.053)	**0** **.** **010**		
NLR	0.999 (0.992–1.007)	0.877		
Ln (NT-proBNP), pg/ml	5.389 (3.146–9.231)	**<0** **.** **001**	4.323 (2.433–7.679)	**<0** **.** **001**
CKMB, ng/ml	1.004 (0.999–1.008)	0.095		
Length of stay, days	1.255 (1.128–1.396)	**<0** **.** **001**		
LVEF, %	0.881 (0.840–0.925)	**<0** **.** **001**		
LVED, cm	2.447 (1.146–5.225)	**0** **.** **021**		
sST2, ng/ml	1.033 (1.020–1.046)	**<0** **.** **001**	1.020 (1.003–1.037)	**0** **.** **022**

Hs-CRP, high-sensitivity C-reactive protein; NLR, neutrophil/lymphocyte ratio; AST, aspartate transaminase; ALT, alanine transaminase; NT-proBNP, N terminal pro-B-type natriuretic peptide; CKMB, creatine kinase isoenzyme MB; LVEF, left ventricular ejection fraction; LVED, left ventricular end diastolic; sST2, soluble suppression of tumorigenicity 2.

Bold values indicate significant *p* < 0.05.

### Receiver operating characteristic

**Figure 2:**
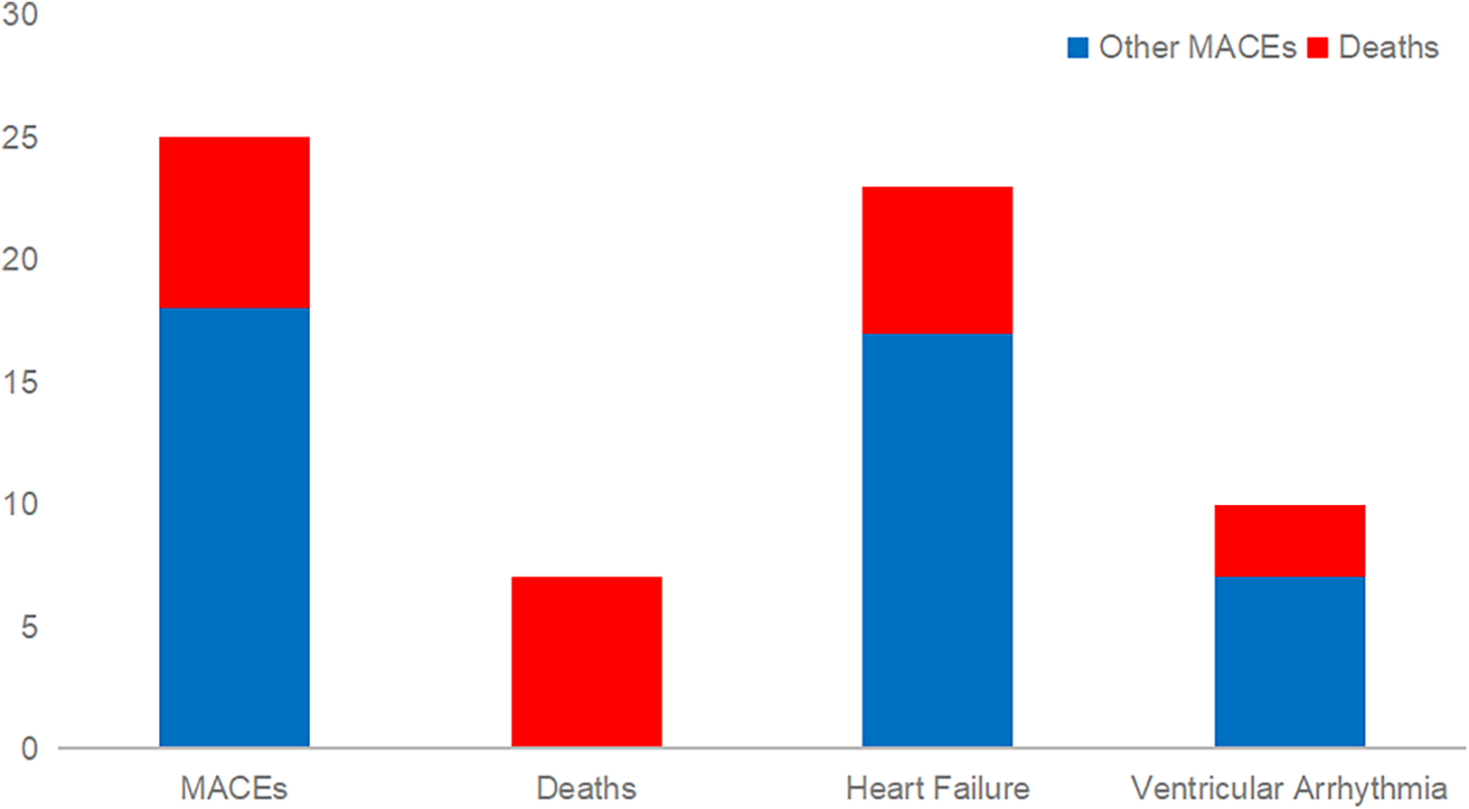
ROC curve analysis association of NT-proBNP and sST2 with the risk of MACEs. NT-proBNP N terminal pro-B-type natriuretic peptide; sST2 Soluble suppression of tumorigenicity 2.

NT-proBNP and sST2 were included in the ROC analysis. ROC analysis for NT-proBNP found an area under the curve (AUC) of 0.96, 95% CI of 0.922–0.998, a cut-off value of 745.5 pg/ml, sensitivity of 0.96, specificity of 0.872, and Youden index of 0.832. ROC analysis for sST2 found an AUC of 0.839, 95% CI of 0.767–0.911, cut-off value of 31.6 ng/ml, sensitivity of 0.88, specificity of 0.718, and Youden index of 0.598. Both NT-proBNP and sST2 showed good predictive efficacy for MACEs (Table 3, Figure 2).

**Table 3:** Receiver operating characteristic.

	AUC	95% CI	Cut-off	Sensitivity	Specificity	Youden index
sST2	0.839	0.767–0.911	31.6	0.88	0.718	0.598
NT-proBNP	0.960	0.922–0.998	745.5	0.96	0.872	0.832

NT-proBNP, N terminal pro-B-type natriuretic peptide; sST2, soluble suppression of tumorigenicity 2.

## Discussion

To our knowledge, this study was the first to investigate the relationship between MACEs and sST2 during hospitalization in pediatric patients with myocarditis. The main finding of this study was that NT-proBNP and sST2 were independent risk factors for MACEs during hospitalization in pediatric myocarditis patients, and both showed good predictive power.

Inflammation can lead to arrhythmogenesis by altering ion channels, ventricular tachycardia (VT) and ventricular fibrillation (VF) are common complications of myocarditis in children and are usually associated with significant hemodynamic impairment (22, 23). In our study, the mortality rate in patients with ventricular arrhythmias was 30%, and patients with ventricular arrhythmias were ten times more likely to die during hospitalization than those without ventricular arrhythmias. This is a similar result to previous studies (24).

Considering the rapidly changing and progressive nature of myocarditis in children, a reliable rapid marker to early identify those at risk would be essential. Previous studies have shown that myocarditis and the inflammatory response are closely linked in adults and children (7, 8). sST2 is an interleukin (IL) receptor family member and can be expressed in various circulating immune cells and cardiac myocytes. sST2 acts as a detrimental “decoy receptor” for circulating IL-33, minimizing the protective effect of IL33 on the cardiovascular system (9, 25). Coronado MJ et al. found that serum sST2 levels were associated with cardiac inflammation in adult patients with myocarditis. Elevated serum sST2 was associated with an increased risk of heart failure in men ≤50 years of age (17). In this study, we found that creatinine, urea, AST, ALT, hs-CRP, NLR, NT-proBNP, CKMB, length of stay, LVED, and sST2 were significantly higher in the MACEs group compared to the No MACEs group, while LVEF was significantly lower. This suggests that patients with myocarditis in combination with MACEs may have a more severe inflammatory response and impairment of organ function. After adjusting for possible risk factors, multifactorial regression analysis showed that NT-proBNP and sST2 were independent risk factors for MACEs during hospitalization in children with myocarditis. Recent studies indicate that sST2, unlike NT-proBNP, does not appear to be affected by renal function or residual diuresis (26, 27). So, the correlation analysis of ST2 with creatinine was conducted (Supplementary Figure S1). Consistent with previous findings, this study discovered no significant correlation between sST2 and renal function, and sST2 was associated with prognosis in children with myocarditis independent of renal function. A previous study has shown that sST2 levels at admission in patients with ICM (inflammatory cardiomyopathy) correlate with the degree of functional left ventricular impairment. In addition, ICM patients with elevated baseline sST2 levels were at higher risk of developing NYHA class III/IV at 12-month follow-up compared to ICM patients with lower baseline sST2 levels (28). In patients with acute myocardial infarction, higher sST2 levels are associated with an increased risk of cardiovascular death and heart failure within 30 days (15). sST2 was a predictor of all-cause and cardiovascular event-related death in patients with chronic heart failure in a meta-analysis by Aimo et al. (29).

In a previous study, Amer, Eslam et al. included 60 children with congestive heart failure as a patient group and sixty age- and sex-matched healthy children as a control group. The results showed that sST2 is a good diagnostic and predictive biomarker for children with congestive heart failure (30). As a traditional cardiac biomarker, NT-proBNP has been extensively reported to be significantly elevated in patients with myocarditis or animal models of myocarditis and is a risk factor for poor prognosis in children with fulminant myocarditis (31–34). An observational study of suspected myocarditis found that higher BNP levels were associated with higher mortality and that elevated BNP levels were independently associated with poor patient prognosis (35). Although CKMB and LVED were elevated and LVEF was reduced in the MACEs group compared to the No MACEs group, they were not independent risk factors for MACEs during hospitalization in children with myocarditis, which is similar to the results of the previous study (35). However, Schultz et al. showed different results regarding LVEF (36). This is controversial and may need to be explored in more studies in the future. Including NT-proBNP and sST2 in the ROC analysis showed that both had reasonable specificity and sensitivity for MACEs during hospitalization in pediatric patients with myocarditis. Previous studies have shown that baseline sST2 is comparable to NT-proBNP in predicting MACEs in ICM cohorts (28). Braunwald E showed that BNP levels were superior to endothelin-1 or norepinephrine as a predictor of death (37). This study found that NT-proBNP appeared to perform more superiorly, which may be related to the predominance of heart failure in the MACEs in this study. More studies should be conducted in the future to explore the superiority of the two biomarkers. Myocarditis in children is an important challenge for clinicians. Our study suggests that sST2 may be a useful marker for the development of in-hospital MACEs in pediatric myocarditis patients. In contrast to NT-proBNP, sST2 is not significantly affected by renal function and may be uniquely valuable in patients with renal insufficiency. Early identification of these high-risk patients by sST2 will perhaps help optimize risk stratification, guide clinical decision-making, and improve prognosis.

## Limitations

This was a single-center retrospective cohort study, and some bias may exist. Second, this study focused on the MACEs during hospitalization, and there is insufficient data on these patients' long-term prognosis. Third, this study only detected the baseline sST2 levels at admission and was not continuously monitored dynamically.

## Conclusion

Elevated levels of NT-proBNP and sST2 were independently associated with major adverse cardiovascular events during hospitalization in children with myocarditis, and both showed good predictive efficacy.

## Data Availability

The raw data supporting the conclusions of this article will be made available by the authors, without undue reservation.

## Supplementary material

Supplementary Figure S1 The correlation analysis of sST2 with creatinine.
