# Supplementary figures and images for: Soluble suppression of tumorigenicity 2 associated with major adverse cardiac events in children with myocarditis
Source: Front Cardiovasc Med. 2024 May 13;11:1404432. doi: 10.3389/fcvm.2024.1404432 (PMC11130408; doi:10.3389/fcvm.2024.1404432)

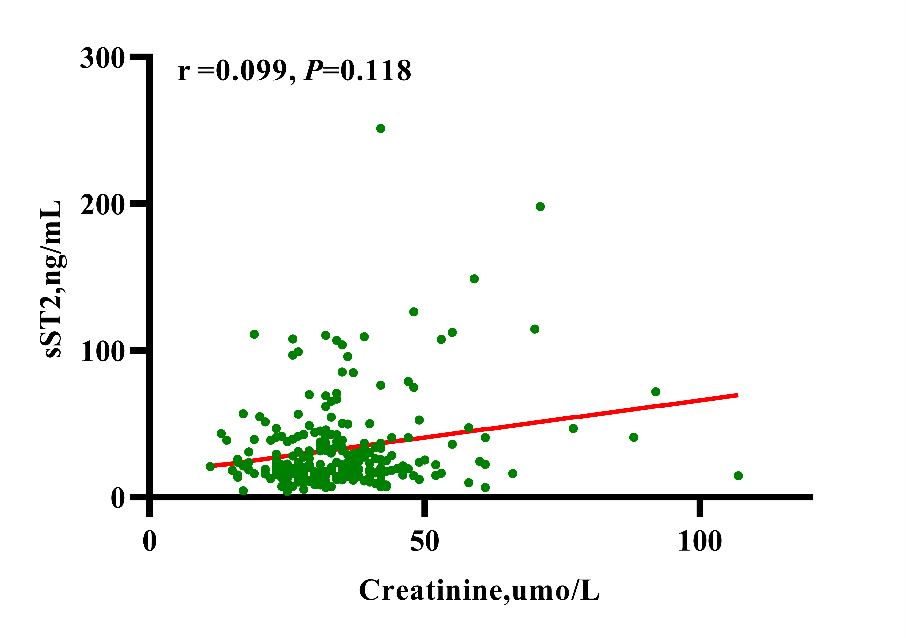

Supplement: Supplementary Figure S1 — The correlation analysis of sST2 with creatinine. [file Image1.tif]
